# Efficacy of Neurorehabilitation Approaches in Traumatic Brain Injury Patients: A Comprehensive Review

**DOI:** 10.3390/life15030503

**Published:** 2025-03-20

**Authors:** Diana Andrei, Alexandra Laura Mederle, Laura Andreea Ghenciu, Claudia Borza, Alexandra Corina Faur

**Affiliations:** 1 Department XVI, Discipline of Medical Rehabilitation, University of Medicine and Pharmacy “Victor Babes”, 300041 Timisoara, Romania; andrei.diana@umft.ro; 2 Department XIV, Discipline of Dermatology, “Victor Babeș” University of Medicine and Pharmacy, 300041 Timisoara, Romania; 3 Department III, Discipline of Pathophysiology, “Victor Babeș” University of Medicine and Pharmacy, 300041 Timisoara, Romania; bolintineanu.laura@umft.ro (L.A.G.); borza.claudia@umft.ro (C.B.); 4 Center for Translational Research and Systems Medicine, “Victor Babeș” University of Medicine and Pharmacy, 300041 Timisoara, Romania; 5 Centre of Cognitive Research in Pathological Neuro-Psychiatry NEUROPSY-COG, “Victor Babeș” University of Medicine and Pharmacy, 300041 Timisoara, Romania; 6 Department I, Disciplone of Anatomy and Embriology, ‘Victor Babes’ University of Medicine and Pharmacy Timisoara, 300041 Timisoara, Romania; faur.alexandra@umft.ro

**Keywords:** traumatic brain injury, TBI, neurorehabilitation, motor rehabilitation, cognitive rehabilitation, virtual reality, VR, telemonitoring, non-invasive brain stimulation, robotic device

## Abstract

Traumatic brain injury (TBI) represents a significant public health issue, causing long-term disabilities and imposing considerable socioeconomic and healthcare challenges. While advancements in acute care have improved survival rates, the demand for effective neurorehabilitation is increasing. This narrative review explores the evidence on neurorehabilitation strategies for TBI, focusing on interventions targeting cognitive, motor, and psychological recovery. A total of 32 studies were included and categorized into six approaches: non-invasive brain stimulation, virtual reality (VR), computer-based training, telerehabilitation, robot-assisted therapy (RAT), and mixed approaches. Non-invasive brain stimulation techniques, such as transcranial direct current stimulation (tDCS) and repetitive transcranial magnetic stimulation (rTMS), showed variable effectiveness in improving cognitive outcomes. VR-based therapies enhanced attention and executive functions, while RAT, such as Lokomat and exoskeletons, improved gait symmetry and functional mobility. Computer-assisted programs demonstrated benefits in rehabilitating social cognition and executive functions. Telerehabilitation and telephone-based treatments provided short-term gains but lacked sustained effects. Overall, cognitive improvements were better described and represented, while several motor improvements lacked consistency. Despite the promising results, significant gaps remain, including heterogeneity in methodologies, small sample sizes, and limited long-term outcome data.

## 1. Introduction

Traumatic brain injury (TBI) is a significant public health concern, described as alterations in brain function resulting from an external force [1]. TBI ranges from mild, with a brief loss of consciousness or confusion, to severe, involving prolonged unconsciousness, amnesia, or long-term neurological impairment. It is a significant cause of fatalities or severe physical and mental dysfunction, resulting in social and economic costs [2]. Globally, TBI incidence is increasing due to urbanization, motorization, and aging populations. Recent research indicates that over 40% of individuals hospitalized with moderate to severe TBI experience long-term effects and chronic disabilities, including headaches, memory loss, irritability, and depression. This condition contributes to approximately 69 million new cases worldwide annually, creating significant socioeconomic and healthcare challenges [3,4]. Few studies have reported age-standardized incidence rates, which are essential for comparing countries with different population structures. However, the available data indicate significant regional variations in the incidence of TBI. Central Europe, Eastern Europe, and Central Asia show notably higher TBI incidence rates compared to other regions, with some of the highest rates observed in Syria, Slovenia, and the Czech Republic [5]. Adults over 65 years old have the highest TBI hospitalization rates, followed by children and teenagers. This pattern indicates an increase in TBI in older adults that is frequently brought on by falls, which raises the likelihood of post-recovery decline and causes more severe cognitive and functional deficits, which might affect personal or social life (walking and feeding difficulties, leading up to the inability to perform self-care) [6,7,8].

TBI is a complex neurological condition with far-reaching consequences beyond physical impairments. In recent years, there has been a growing body of research focusing on cognitive impairment and rehabilitation [9,10,11]. However, despite the abundance of studies on cognitive rehabilitation [10], translating this evidence into routine clinical practice remains challenging [12]. Cognitive and emotional aspects—such as memory impairment, concentration difficulties, and executive dysfunction—are often under-addressed in clinical settings that prioritize physical recovery. Traditional interventions such as balance and strength training and functional electrical stimulation may be less effective in these cases, as they do not inherently enhance patient motivation or engagement. However, innovative approaches like robot-assisted therapy (RAT) and virtual reality (VR)-based rehabilitation have shown promise in overcoming these barriers by increasing patient motivation, engagement, and therapy adherence, ultimately improving rehabilitation outcomes [13]. Emotional disturbances, including melancholy, anxiety, and mood swings, can further affect motivation and participation in rehabilitation programs aimed at improving gait [14].

Cognitive and psychological recovery varies widely among TBI patients. While many individuals show significant improvement within the first year post-injury, residual deficits may persist, particularly in severe cases. The clinical diversity and broad spectrum of TBI make both early therapy and rehabilitation extremely difficult [15]. However, the overall survival rate for individuals with severe TBI has risen due to advancements in healthcare technology for initial recovery and acute treatment, and so has the demand for long-term treatment [16].

Neurorehabilitation is an interdisciplinary approach aimed at improving recovery, functionality, and quality of life for individuals with neurological disorders or injuries. Additionally, the currently available information suggests that rehabilitation is more effective when it is started shortly after injury and maintained over time [17]. However, only a small percentage of those impacted received rehabilitation that targeted cognitive and psychological impairments, even though about one-third of TBI patients reported having deficits [18]. The CENTER-TBI trial included 1206 TBI patients who experienced moderate to severe impairment 6 months after the event, with about 90% of patients requiring rehabilitation. However, a mere 30% obtained inpatient rehabilitation, and only 15% obtained outpatient rehabilitation [19].

The development of advanced technologies has created new opportunities to help TBI sufferers regain their mobility and improve their general quality of life [20]. Growing research indicates that patients with neurological diseases could benefit from robotics, virtual reality, and other technologies, despite certain obstacles that may limit the application of novel technology in clinical practice [21,22].

The aim of this review is to provide an overview of current neurorehabilitation approaches for patients with TBI, focusing on their cognitive, motor, and psychological outcomes. Innovative technologies in the field of neurorehabilitation encourage repetition, longer therapy sessions, and increased motivation. These factors promote neuroplasticity, which in turn leads to functional recovery and, consequently, an improved quality of life. By examining innovative interventions such as non-invasive brain stimulation, virtual reality-based therapies, telerehabilitation, RAT, and computer-assisted training, this review offers insight into the range of rehabilitation options available for TBI patients.

## 2. Materials and Methods

### 2.1. Study Design and Scope

This narrative review aims to provide a comprehensive overview of the existing literature on neurorehabilitation interventions for TBI, focusing on cognitive, motor, and psychological recovery. The review summarizes findings from primary research studies, clinical trials, and case reports on non-invasive brain stimulation, virtual reality, computer-based interventions, telerehabilitation, robotic therapy, and mixed approaches.

### 2.2. Literature Search Strategy

Two independent authors identified relevant studies by searching for articles available in scientific journals. The sources included multiple electronic databases such as PubMed, Scopus, Google Scholar, and Web of Science, as well as references from key articles and reviews. We used the following search terms: “traumatic brain injury”, or “TBI”, and “neurorehabilitation” followed by “virtual reality”, or “VR”, or “robot”, or “innovative approach”, or “exoskeleton”, or “computer-based”, or “non-invasive brain stimulation”, or “telemonitoring”, or “telephone-based” for studies published in the last ten years (last search: 20 January 2025). Boolean operators were used to combine the terms (“AND”, “OR”). Additionally, article titles and abstracts, containing search term synonyms were also screened. The initial selection involved screening titles and abstracts for eligibility. Full-text articles were then assessed according to the inclusion and exclusion criteria. Articles were included if they (1) investigated at least one group of patients with TBI diagnosis (with or without a control group), (2) described interventions for TBI rehabilitation, (3) reported or measured outcomes related to cognitive, motor, or psychological recovery, (4) were published in English, and (5) were published in a peer-reviewed journal. Studies were excluded if they (1) assessed groups of patients with other diagnoses, (2) were abstracts, reviews, letters to editor, or (3) were published in other languages. Experimental studies, case reports, and feasibility studies with an experimental component were considered. The final selection included primary research studies, clinical trials, feasibility studies, and case reports that specifically addressed neurorehabilitation tools and their effectiveness in cognitive, motor, and psychological recovery for TBI patients.

### 2.3. Data Extraction

Data were manually extracted from included articles and summarized based on author and year of publication, study design, intervention type, sample size, duration and intensity of the intervention, and key findings. Specific outcomes such as improvements in cognitive scores, motor function, or quality of life measures were highlighted where available.

Given the heterogeneity of study designs, interventions, and outcome measures, a direct comparison through the meta-analysis of all neurorehabilitation tools is not feasible; therefore, a narrative synthesis approach was used approach allowed for the descriptive comparison of interventions and their reported effectiveness without conducting formal meta-analytical techniques. Common themes, gaps in knowledge, and patterns between treatments were discovered and discussed.

## 3. Neurorehabilitation Approaches

### 3.1. Summary of Selected Studies

**Figure 1:**
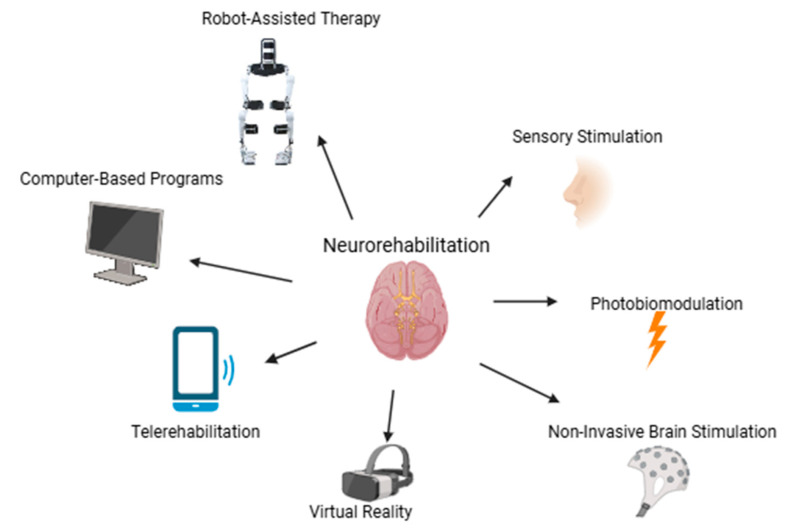
Graphical illustration of neurorehabilitation approaches for TBI patients. Created with https://www.biorender.com/ (accessed on 2 March 2025).

A total of 40 studies were included in this review since they matched our inclusion criteria. Given the many techniques and approaches they described, the results were separated into eight categories: twelve studies focused on non-invasive brain stimulation (out of which two were case studies), six on virtual reality interventions (out of which two were case studies), three on computer-based interventions, four on telerehabilitation, six on robotics (out of which two were case studies), six on laser therapy (out of which two were case studies), two on sensory stimulation, and one on a mixed approach. The majority of the studies, regardless of the tool used, described at least one cognitive outcome. The studies included in this review can be found in Supplementary Files S1. We excluded case reports and feasibility studies from this table because they either lacked statistical power or assessed the practicality of interventions rather than their efficacy. These were, however, included in the narrative review. Figure 1 represents an overview of non-invasive approaches described in this review.

### 3.2. Non-Invasive Brain Stimulation

Non-invasive brain stimulation (NIBS) approaches have been developed as novel tools for neurorehabilitation (Table 1). These treatments attempt to modify brain activity and induce neuroplasticity, therefore aiding in the recovery of physical, cognitive, and psychological functions in TBI patients [23,24]. A systematic review by Korupolu et al. highlighted the current state of research on vagus nerve stimulation (VNS) for motor recovery in animal models and humans. Among the 19 studies identified, most were conducted in animal models of stroke, with only one study involving TBI and none in human TBI populations. However, the TBI study in animals demonstrated the potential of VNS paired with rehabilitation to enhance motor recovery compared to rehabilitation alone [25]. Chen et al. suggested that rTMS combined cognitive training is a promising strategy to improve cognition and functional outcomes in TBI patients. Their systematic review and meta-analysis demonstrated that rTMS combined with cognitive training significantly improved cognitive function in patients with post-traumatic brain injury cognitive disorder. Fourteen studies showed that rTMS + CT improved global cognitive outcomes and showed enhancements in memory and daily living abilities. Additionally, rTMS + CT reduced P300 latency, reflecting improved cognitive processing speed [26].

The study by Schiff et al. explored the use of deep brain stimulation targeting the central lateral thalamus and its associated dorsal tegmental tract in patients with moderate-to-severe TBI. All participants showed significant improvements in executive function, with an average 31.7% improvement in Trail Making Test-Part B performance and enhancements in attention and quality-of-life measures. Electrophysiological recordings confirmed cortical activation, suggesting that this technique facilitates recovery in frontal-striatal networks [27]. Another study introduced the brain–machine interface (nrBMI) which leverages high-gamma neural signals acquired through EEG or hemicraniectomy-adapted hEEG to decode continuous, proportional motor intent, providing precise haptic feedback to simulate sensory experiences of successful motor execution. Participants achieved successful control, with TBI participants using high-gamma signals demonstrating an average success rate of 62% across trials. Over time, participants exhibited learning, with improved performance [28].

Eight articles, out of which two were case studies, discussed the possibility of using tDCS as a tool in neurorehabilitation. In the study by Secco, an experimental group received tDCS over the dorsolateral prefrontal cortex combined with divided attention (DA) training. The group showed significant post-treatment improvements in reaction times and omission errors during DA tasks, which were stable at a one-month follow-up. Apathy scores also improved in the experimental group, but no significant changes were observed in depression scores [29]. In another study, the tDCS group demonstrated significant improvements in depression, anxiety, and post-concussive symptoms, as well as in complex attention and executive function tasks. MRI analysis revealed that the active tDCS group showed an increase in cerebral perfusion in the right inferior frontal gyrus compared to reductions observed in the control group [30]. Li et al. employed tDCS paired with a Stop Signal Task, which is used to assess cognitive and neural changes in moderate-to-severe TBI patients. Anodal tDCS improved the response inhibition in healthy controls but not in TBI participants [31]. Studies on tDCS also showed improved verbal retrieval and episodic memory performance in the experimental group compared to controls [32,33]. O’Neil-Pirozzi et al. showed that both the control and TBI groups experienced significant improvement in word recall following anodal tDCS compared to cathodal and sham conditions, with no significant changes in the latter two. The P300 amplitude increased significantly in the TBI group only after anodal tDCS, with no significant effects on latency or EEG power across conditions [34].

Two additional studies described repetitive transcranial magnetic stimulation (rTMS), a method used to induce neuroplastic changes in the brain, applied for the treatment of neurological diseases, such as stroke, epilepsy, and movement disorders [35]. Lee et al. applied low-frequency rTMS to the right dorsolateral prefrontal cortex in 13 patients with moderate TBI. The experimental group showed significant improvements, including a 29.29% reduction in depression scores (MADRS), a 6.25% decrease in Trail Making Test (TMT) time, and a 12.64% decrease in Stroop Color Word Test (SCWT) time. In contrast, the control group showed minimal changes in all measures [36]. Another study found no significant improvements in cognitive function in the real rTMS group compared to the sham group [37].

Two case-control studies described tDCS in the neurorehabilitation of TBI patients. Both studies described the effectiveness of tDCS in facilitating cognitive recovery in individuals with moderate-to-severe TBI, but they differ in several key aspects. The first study used high-definition tDCS (HD-tDCS) targeting the pre-supplementary motor area (pre-SMA), delivering 10 sessions to a 39-year-old woman, leading to improvements in verbal fluency, naming, working memory, and executive functions, with gains persisting for up to 14 weeks [38]. In contrast, the second study involved 20 sessions of remotely supervised tDCS (RS-tDCS) combined with cognitive training, targeting broader cognitive domains such as attention, semantic fluency, and processing speed in a 29-year-old man [39].

**Table 1:** Non-invasive brain stimulation in TBI neurorehabilitation.

Study	Sample Size Control/Experimental Group	Method	Cognitive Outcomes	Duration	Motor Outcomes	Other
Flint et al., 2023 [28]	3/3	High-γ/μ–β signals	Motor planning, learning, and adaptability improved in the experimental group.	N/A	Motor output (force production), thumb compliance, and physical execution of tasks improved.	Differences in success rates between participants (T1: 28% vs. T2: 99%)
Quinn et al., 2020 [30]	14/10	Computerized executive function training combined with tDCS	Participants demonstrated significant improvements in depression, anxiety, post-concussive symptoms, complex attention, and executive functions from baseline to post-treatment visits (*p* < 0.01).	30-min sessions, 10 weekdays	N/A	Global cerebral blood flow decreased significantly from baseline to post-treatment visits (*p* = 0.02), with no significant differences between mild and moderate TBI participants.
Lee et al., 2018 [36]	6/7	Neurodevelopmental therapy and rTMS intervention	The experimental group demonstrated significant improvements in post-intervention scores for the Montgomery-Asberg Depression Rating Scale (MADRS), Trail Making Test (TMT), and Stroop Color Word Test; the control group showed no significant changes in these measures.	30-min sessions 5 days per week for 2 weeks	N/A	N/A
Li et al., 2019 [31]	24/31	tDCS sessions	Anodal tDCS did not improve response inhibition in TBI patients; control showed significant improvement in SSRT under anodal tDCS. TBI participants showed no significant improvement in Stop Signal Delay under any stimulation condition.	Single visit, three stimulation conditions	N/A	N/A
Motes et al., 2019 [32]	6/8	HD-tDCS sessions	Significant differences between the active and sham groups in the changes in the total score of the Rey Auditory Verbal Learning Test (RAVLT), the time taken for the Delis–Kaplan Executive Function System (DKEFS) Inhibition test, and the time taken for the DKEFS Inhibition/Switching Test.	Ten sessions of 20 min of 1 mA anodal HD-tDCS	N/A	N/A
Neville et al., 2019 [37]	13/17	rTMS was applied using a magnetic stimulator (MagPro X100).	N/A	5 s of rhythmic high-frequency daily for 10 sessions	No consistent improvement in executive function of controls versus patients.	N/A
O’Neil-Pirozz et al., 2017 [34]	4/4	tDCS sessions	For the TBI group, word recall increased in the anodal condition for all participants (+3 to +6 words) and in the sham condition for two participants.	Three 90-min sessions, a minimum of 48 h apart	N/A	P300 latency increased across all conditions for the TBI group.
Sacco et al., 2016 [29]	16/16	tDCS stimulation (HDCstim device)	Within the experimental group, significant improvements were observed between pre-training and post-training, with faster reaction times (*p* = 0.004) and fewer omission errors; the control group did not exhibit any significant changes; there was borderline improvement in attention performance (*p* = 0.057) within the experimental group, although no significant changes were found in visual-spatial abilities, semantic fluency, working memory, or long-term memory.	Ten sessions, with each session including 20 min of tDCS stimulation followed by 30 min of cognitive training	N/A	N/A

Legend: N/A—not applicable; rTMS—transcranial magnetic stimulation; TBI—traumatic brain injury; tDCS—transcranial direct current stimulation.

### 3.3. Virtual Reality

VR has become known as a promising approach to neurorehabilitation following TBI [40]. VR offers unique opportunities for cognitive, motor, and social rehabilitation because it generates realistic, dynamic, and personalized environments. VR-based interventions can simulate real-life situations that promote skill development, such as balance training, memory enhancement, and executive function improvement in a safe and stimulating setting [41]. According to studies, VR can enhance neuroplasticity by combining sensory feedback, task repetition, and real-time performance monitoring. Furthermore, VR’s gamified components can boost patient motivation and compliance with therapy [41,42]. VR, when combined with regular rehabilitation treatments or neuromodulatory methods such as tDCS, may improve treatment outcomes [43,44]. Table 2 highlights studies regarding VR in patients with TBI.

Liu et al. observed individuals who used a custom glove with VR-based augmented sensory feedback during a grasp-and-place task. TBI participants showed significant improvements in task performance (reduced motion pathlength and completion time) and increased neural activity (EEG alpha and beta power) with multimodal feedback (audio and visual) compared to unimodal. Additionally, neural–muscle coordination improved with multimodal feedback [45]. Another study compared virtual reality-based attention training using the VRRS system to conventional attention training. While both approaches improved cognitive and mood outcomes, the first group showed significantly greater improvements in attention-related subdomains, including visual attention, task switching, and speed of processing, as well as reductions in depressive symptoms [46].

The NeuroDRIVE intervention, a virtual reality-based driving rehabilitation program, was tested on 27 participants with a history of TBI. While VR driving performance did not show significant differences between groups, the intervention group demonstrated significantly greater improvements in working memory (*p* = 0.004) and visual search/selective attention (*p* = 0.01). Additionally, physical functioning scores (*p* < 0.05) improved more in the intervention group. However, other cognitive measures, neurobehavioral symptoms, and quality of life indicators did not show significant differences between groups [47]. Additionally, when comparing the effects of VR home exercise programs (HEP) to traditional HEP on balance and participation outcomes in individuals with chronic TBI, no significant between-group differences were observed in any of the balance measures or secondary outcomes, including balance confidence and community participation [48].

Two case studies evaluated the effectiveness of VR-based interventions for improving balance and related functions in patients with TBI. Both interventions significantly improved dynamic balance and functional mobility in TBI patients, with the CAREN system normalizing vestibular function and enabling a return to active duty, while the Xbox Kinect intervention enhanced cardiovascular fitness and community mobility [49,50].

**Table 2:** Virtual reality applications in TBI neurorehabilitation.

Study	Sample Size Control/Experimental Group	Method	Cognitive Outcomes	Duration	Motor Outcomes	Other
De Luca et al., 2023 [51]	10/10	Standard cognitive rehabilitation/Virtual Reality Rehabilitation System	The VR group demonstrated significant improvements in cognitive functions, emotional well-being, and coping strategies compared to the standard rehabilitation group, particularly in attention, executive functioning, and problem-solving.	3 months standard neurorehabilitation (6 weekly sessions of 60 min), 3 months advanced rehabilitative (3 weekly sessions of 60 min)	N/A	N/A
De Luca et al., 2022 [46]	15/15	Standard cognitive rehabilitation/Virtual Reality Based-Attention Processes Training (VB_APT)	The experimental group showed significantly greater improvements in global cognition, attention, and depression compared to conventional rehabilitation and significant improvements in executive, visual-spatial, and attention subtests.	First phase: 3 times a week for 8 weeks Second phase: 24 sessions of 60 min each, 3 times a week for 8 weeks	N/A	N/A
Ettenhofer et al., 2019 [47]	6/11	VR driving simulator NEUROdrive	Intervention group had greater improvement in in working memory (*p* = 0.004) and visual search/selective attention (*p* = 0.01). No significant differences between groups in other cognitive measures.	Six 90-min sessions, 4 weeks	No significant change between groups (*p* > 0.05) in VR tactical and operational scores. Scores remained “average” at both time points in the intervention group.	Greater improvement in intervention group (*p* < 0.05) for physical functioning. No significant difference (*p* > 0.05) for mental functioning.
Liu et al., 2023 [45]	13/7	Participants used instrumented glove on their dominant hand and performed a grasp-and-place maneuver; they also experienced VR environment.	N/A	One single session, three blocks of trials	Multimodal feedback was effective in enhancing neural activity and improving motor performance in TBI participants, as demonstrated by increased EEG power, improved motion pathlength, and greater EMG-EEG coherence. Neurotypical participants responded better to unimodal feedback, with faster task completion and reduced EMG coherence.	N/A
Teterfiller et al., 2019 [48]	32/31	Xbox Kinect games	N/A	3–4 times per week for 12 weeks, lasting for 30 min	No statistically significant difference between the two groups; both treatment groups showed improved balance responses to these therapies.	N/A

Legend: EEG—electroencephalography; EMG—electromyography; VR—virtual reality.

### 3.4. Computer-Based Programs

Computer-based programs are becoming increasingly significant in TBI neurorehabilitation, providing unique techniques for addressing cognitive, affective, and motor challenges. These programs offer a controlled and adaptable setting for addressing specific deficiencies such as memory, attention, executive functioning, and social cognition [52]. One significant advantage of computer-based programs is their capacity to personalize therapy. Adaptive algorithms change the intensity of tasks in real time, ensuring that patients are constantly being challenged but not overburdened. These systems also support the use of multimodal input, such as auditory, visual, and tactile cues, to improve learning and engagement. Furthermore, the remote delivery of some computer-based therapies improves access for patients who are unable to attend in-person rehabilitation sessions [53]. Table 3 highlights research studies focused on computer-based programs in the rehabilitation of patients with TBI.

Rodríguez-Rajo et al. demonstrated that a computerized social cognition (SC) module significantly improved SC measures in patients with moderate-to-severe TBI in the subacute phase, particularly for the Reading the Mind in the Eyes Test (RMET). Combined treatment (SC + non-SC) proved more effective for SC rehabilitation compared to non-SC treatment alone [54]. Another study evaluated the effectiveness of computer-assisted cognitive rehabilitation (CACR) in improving cognitive function in patients with cognitive impairments (CI) following TBI. The results indicated significant improvements in cognitive and functional outcomes for the experimental group compared to the control group. Additionally, the experimental group showed notable gains in social cognitive ability, self-care skills, and overall functional independence. The study concluded that CACR is an effective tool for cognitive rehabilitation in TBI patients, promoting recovery, enhancing quality of life, and achieving better rehabilitation outcomes compared to traditional methods [55].

A qualitative study employed a user-centered design approach to develop and test a Brain–Computer Interface (BCI) for cognitive rehabilitation in individuals with moderate-to-severe TBI. Participants completed two cognitive tasks (“Find a Category” and “Memory Card Game”) through the BCI system. Feasibility testing showed accuracy rates of 91.87% for the control group and 78.13% for TBI participants, with all exceeding the 70% usability threshold. Despite technical challenges, the system demonstrated potential for improving cognitive engagement and rehabilitation accessibility for TBI patients [56].

**Table 3:** Computer-based training in TBI neurorehabilitation.

Study	Sample Size Control/Experimental Group	Method	Cognitive Outcomes	Duration	Motor Outcomes	Other
Liu et al., 2021 [55]	30/30	Computer-assisted cognitive rehabilitation (CACR system)	Significantly better cognitive scores in social cognitive ability, self-care ability, sphincter control, and comprehensive ability compared to the control group (*p* < 0.05).	N/A	N/A	N/A
Rodriguez-Rajo et al., 2024 [54]	26/28	Computerized tasks module designed for the rehabilitation of social cognition	Experimental group showed better results for almost all measures. Improved ability to recognize facial emotions in the control group. Experimental group demonstrated better ability to recognize emotions or mental states from eyes post-treatment compared to the control group.	Weekly sessions	N/A	N/A

### 3.5. Telerehabilitation

Telerehabilitation in TBI is a fast-growing strategy that uses technology to deliver rehabilitation treatments remotely, eliminating geographical and logistical constraints to care. It comprises a wide range of interventions, including cognitive, physical, speech, and occupational therapy, which are provided via telecommunication platforms such as video conferencing, smartphone apps, or web-based programs [57]. Telerehabilitation allows for the ongoing assistance and monitoring of TBI patients, particularly those in remote or underserved areas. It has demonstrated potential in enhancing cognitive outcomes such as memory, attention, and executive function through structured cognitive training regimens. Physical therapy, including balance and motor skill training, has also been successfully applied using virtual environments and motion-sensing devices [58]. Furthermore, speech treatment for communication problems can be delivered in real time with therapists [59]. Table 4 highlights studies focused on telerehabilitation for patients with TBI.

De Luca et al. assessed the feasibility and usability of a telerehabilitation system, the Virtual Reality Rehabilitation System (VRRS), for patients with severe TBI and their caregivers during a pre-discharge training phase. The study found promising results: both patients and caregivers demonstrated positive motivation, reflected by high Intrinsic Motivation Inventory scores, and good usability scores on the System Usability Scale. Younger patients showed slightly higher usability scores than older ones. Training caregivers alongside patients enhanced system usability and facilitated a smoother transition to independent telerehabilitation at home [60]. Raso et al. also evaluated the effectiveness of a telemonitoring system for patients with severe TBI requiring long-term care, comparing it to traditional long-term hospital stays. The telemonitoring system utilized videoconferencing, wearable devices, and remote consultations with caregivers trained to assist in daily care tasks. Over a 4-year follow-up, both groups showed similar clinical outcomes in cognitive function, responsiveness, and medical complications. However, the telemonitoring group demonstrated a trend toward fewer complications, such as bedsores and infections, and significantly reduced care costs [61].

Two studies investigated the effects of Problem-Solving Treatment (PST), a structured intervention involving biweekly phone calls to address personal problems, compared to an education-only (EO) group receiving informational brochures. The first study examined psychological distress and post-concussive symptoms (PCS), finding that PST improved psychological distress at 6 months but had no significant effect on PCS, with benefits fading by 12 months. The second study focused on sleep quality, showing that PST significantly improved sleep duration, latency, and efficiency at 6 months, though these gains did not persist at 12 months [62,63].

**Table 4:** Telerehabilitation in TBI rehabilitation.

Study, Year	Sample Size Control/Experimental Group	Method	Cognitive Outcomes	Duration	Motor Outcomes	Other
Bell et al., 2017 [62]	178/178	Telephone-Delivered Problem-Solving Treatment	Experimental group had short-term benefits for psychological distress, sleep quality, depression, PTSD symptoms, and physical health compared to control group (*p* < 0.05). However, these improvements were not sustained at 12 months.	Baseline, 6 months, 12 months	N/A	Participants in the experimental group reported higher satisfaction with the intervention and perceived it as more helpful in addressing their challenges.
Raso et al., 2021 [61]	11/11	Scheduled videoconferences patient-clinical unit; wearable monitoring devices	Higher mortality in the LSH program (36%) compared to the telemonitoring group (18%). Bedsores (18% vs. 0%) and infections (36% vs. 18%) were more common in the LSH group, but differences were not significant. No significant differences between groups in neuropsychological scores.	N/A	4-year intervention period, with monthly consultations for the telemonitoring group	Lower daily health care costs
Vuletic et al., 2016 [63]	178/178	Control group: mailing of educational brochures Experimental group: telephone calls	-	Biweekly over a 6-month period	N/A	Experimental group showed significant improvements in overall sleep quality, sleep duration, latency, and habitual sleep efficiency at 6 months. Improvements in sleep were not sustained at 12 months.

Legend: PTSD—post-traumatic stress disorder.

### 3.6. Robot-Assisted Therapy

#### 3.6.1. Robot-Assisted Motor Therapy

Programs for neurorehabilitation for patients recovering from TBI are increasingly incorporating robotic devices. Through high-intensity, repeated, and task-oriented exercises, these cutting-edge devices support neuroplasticity and early mobilization and gait training [64]. Studies explicitly focusing on robotic neurorehabilitation for TBI patients are rather rare, despite the promising applications. Standardized robotic therapy regimens that are applicable to all patients are difficult to design since TBI involves a wide range of injury severities and affected brain areas. Due to their more consistent and predictable patterns of disability, diseases like stroke have historically been the main focus of robotic rehabilitation research [65]. Table 5 highlights the studies focused on robot-assisted motor therapy for patients with TBI.

Three studies included in the review utilized Lokomat as a tool for rehabilitation. Esquenazi et al. investigated 22 individuals with chronic TBI who were assigned to one of three locomotor training groups: Lokomat RAT, partial body weight–supported treadmill training (PBWSTT), or G-EO end-effector robotic training. Significant gains in self-selected walking velocity (SSV) were noted in all groups during the research. Furthermore, the maximal walking velocity (MV) of the Lokomat and PBWSTT groups increased significantly, whereas the G-EO group did not exhibit a statistically significant change in MV. The groups did not differ significantly in terms of changes in gait symmetry. Interestingly, fewer employees were needed for the Lokomat than for the other approaches, indicating possible benefits in clinical settings [66]. Maggio et al. compared Lokomat Pro, equipped with VR, to Lokomat Nanos, without VR. While both groups reported increases in mood and physical well-being, only the Lokomat Pro with VR group demonstrated significant improvements in global cognitive functions, executive functions, attention processes, and general quality of life [67]. A study of four male patients with disorders of consciousness caused by TBI assessed robotic-assisted gait training with the Lokomat system. All individuals had increased responsiveness, with three of them advancing to overground walking before discharge [68]. These results show the adaptability of Lokomat in meeting both motor and cognitive rehabilitation goals in individuals with TBI.

Two case studies highlight the use of robotic exoskeletons for gait rehabilitation in young male TBI patients, demonstrating improvements in gait symmetry and motor control. The first case demonstrated notable gains in functional independence and joint control, though walking speed and step length decreased without the exoskeleton. Similarly, the second case reported enhanced symmetry and reduced variability in movement patterns but with reduced walking speed and distance [69,70].

**Table 5:** Robot-assisted motor therapy in TBI neurorehabilitation.

Study, Year	Sample Size Control/Experimental Group	Method	Cognitive Outcomes	Duration	Motor Outcomes	Other
Esquenazi et al., 2017 [66]	7/8/7 (all experimental l: G-EO system/Lokomat/PBWSTT groups)	G-EO; Lokomat; manual assisted BWSTT	N/A	18 sessions of gait training for 6 to 8 weeks, generally 3 times per week. Each session lasted up to 75 min.	Functional mobility improved significantly in the G-EO and PBWSTT groups.	All three interventions (G-EO, Lokomat, and PBWSTT) significantly improved self-selected velocity, but only Lokomat and PBWSTT improved maximal velocity; improvements in the stroke impact scale mobility domain were seen only in the Lokomat and PBWSTT groups.
Maggio et al., 2019 [67]	28/28	Lokomat Pro, equipped with a VR screen/Lokomat Nanos, without VR	Significant improvement in global cognitive function, cognitive flexibility and shifting skills, selective attention, and visual search for experimental group. Both groups showed significant improvements in mood and well-being.	40 one-hour sessions (8 weeks, 5 times/week)	Experimental group showed significant improvement in executive functions. Both groups showed significant improvement in physical well-being.	Experimental group showed significant improvement in overall quality of life.

Legend: G-EO—end effector robot; PBWSTT—partial body weight-supported treadmill training.

#### 3.6.2. Robot-Assisted Cognitive Therapy

The Pepper robot has been modified to detect and react to patients’ facial expressions in the context of neurorehabilitation for TBI patients. This improves social interactions and aids in therapeutic procedures. In a study by Ilyas et al., a specific deep-trained model called TBI-FER was presented. Its purpose was to use facial expression recognition (FER) to properly identify the emotions of TBI patients. This concept was incorporated into the Pepper robot, which allows it to perceive and react to patients’ emotional cues to help with rehabilitation exercises. The performance of the TBI-FER model was compared with Pepper’s integrated FER capabilities in order to assess its efficacy. According to the findings, the TBI-FER model performed better at identifying the six fundamental emotions—neutral, happy, angry, sad, tired, and surprised—especially when deciphering the complex and sometimes subtle facial expressions of TBI patients. For example, Pepper’s built-in system detected neutral expressions with 42% accuracy, but the TBI-FER model identified them with 88% accuracy [71].

### 3.7. Photobiomodulation

Photobiomodulation (PBM) is an emerging therapy for TBI that uses red and near-infrared light to stimulate cellular repair, reduce neuroinflammation, and enhance mitochondrial function. Lasers, light-emitting diodes (LEDs), and other light sources, typically with wavelengths between 400 and 1100 nm, can be used to perform PMB, promoting neuroprotection and neurogenesis [72]. Studies suggest that PBM can improve cognitive function, reduce post-TBI depression, and enhance motor recovery. Its non-invasive nature and minimal side effects make it a promising adjunctive treatment in neurorehabilitation for TBI patients. Table 6 highlights the studies focused on photobiomodulation for patients with TBI.

Lin et al. found that PBM reduced cerebellar diaschisis in TBI patients by improving cerebellar perfusion. Although there was no significant improvement in cognitive function compared to standard physical therapy, PBM showed potential in promoting brain perfusion and reducing secondary injury associated with oxidative stress and mitochondrial dysfunction [73]. The research study of Carneiro et al. provides further evidence for PBM-induced changes in patients with chronic TBI. Neuropsychological evaluation demonstrated improvements in executive function, memory, and response time in several tests, while depression scores decreased slightly after PBMT, and anxiety scores showed minimal changes but remained in the mild-to-moderate range [74]. Henderson et al. also reported significant improvements in depressive symptoms in 92% of the patients included in the study [75]. Other studies have also shown significant improvements in cognitive performance, including gains in memory, concentration, processing speed, and sleep quality [76].

In a case study, after 8 weeks of PBM therapy, the subject showed improvements in verbal learning, executive function, attention, and processing speed. His headaches improved, with the Headache Impact Test (HIT-6) score decreasing from 76 to 70. At a 14-month follow-up, the subject reported sustained improvement in headaches (HIT-6 score: 50), continued to use the PBM devices intermittently, and experienced no further concussions [77]. Two adults suffering from TBI have been included in a case series. After 12 weeks of PBM treatments, significant improvements were observed in both of them. Fatigue, pain, sleep, and mood-cognitive symptoms all improved, leading to a substantial reduction in their overall Symptom Severity Index [78].

**Table 6:** Photobiomodulation therapy in TBI neurorehabilitation.

Study, Year	Sample Size Control/Experimental Group	Method	Cognitive Outcomes	Duration	Motor Outcomes	Other
Carneiro et al., 2019 [74]	10 (only experimental group)	Transcranial PBM	Sustained gains in visuospatial ability and planning, improved processing speed and divided attention, modest improvement in inhibition and selective attention	18 sessions, delivered three times per week for six weeks.	N/A	Improved cerebral bloodflow
Henderson et al., 2017 [75]	39 (only experimental group)	Multi-Watt Near-Infrared Phototherapy	N/A	Each session lasted 30 min, with 9–12 min of application per target area (8 to 34 sessions/participant).	N/A	Significant improvement in mood and reduced depression symptoms, reduction in fatigue, and enhanced overall well-being
Hipskind et al., 2018 [76]	12 (only experimental group)	Pulsed Transcranial Red/Near-Infrared Light Therapy Using LED	Significant improvement in symbol search, coding, and processing speed; six of the 15 neuropsychological scales showed significant improvement (*p* < 0.05), particularly in verbal memory and processing speed.	20 min per session, 3 times per week for 6 weeks (total of 18 sessions)	N/A	N/A
Lin et al., 2023 [73]	15/15	Intravenous PBM	Improved short-term memory and attention, reduced confusion and disorganized behavior	60 min session performed on weekdays over two consecutive weeks for each of the three courses	No significant change in motor outcomes	N/A

Legend: PBM—photobiomodulation.

### 3.8. Sensory Stimulation

Sensory stimulation is a non-invasive and simple therapeutic approach used in the rehabilitation of patients with TBI, especially those in a minimally conscious or vegetative state [79]. This technique includes the controlled presentation of stimuli that target several sensory modalities—auditory, visual, tactile, olfactory, and proprioceptive—with the purpose of improving neuronal responsiveness, boosting cognitive recovery, and increasing arousal [80]. The simultaneous use of multiple sensory channels can have a profound impact on recovery, particularly in individuals with impaired consciousness. This integrated sensory approach activates different neural pathways, fostering a more holistic recovery process. The combined effects positively influence cognitive functioning, emotional regulation, and autonomic processes [81,82]. The technique can be effectively facilitated by nurses, therapists, and family members. Studies have shown that incorporating an affective component into a family-centered stimulation strategy improves consciousness in comatose patients [83]. Furthermore, these data suggest that sensory stimulation provided by family members resulted in improved consciousness and cognitive function [84]. Furthermore, personalized sensory stimulation, an advanced approach designed to optimize rehabilitation in patients by tailoring stimuli to individual cognitive and emotional profiles, provides notable advantages over conventional methods by utilizing recent discoveries on residual cognitive function and brain connectivity. It employs structured, meaningful, and multisensory stimuli, incorporating emotionally and autobiographically relevant content to enhance attention and neural activation. Furthermore, it can integrate covert response detection and task-based engagement, creating a more interactive rehabilitation process [85]. Table 7 highlights the studies focused on sensory stimulation for patients with TBI.

The study by Salmani et al. [83] assessed the effects of family-centered affective stimulation on TBI patients. The intervention involved structured activities designed to engage patients emotionally and cognitively through family interaction. The results revealed significant differences in the Glasgow Coma Scale (GCS) scores among the groups at various time points. The intervention group showed a notable increase in GCS scores over time (*p* < 0.001). The placebo group also exhibited an increase in GCS scores, but their improvement was less pronounced compared to the intervention group. The findings also indicated that patients in the intervention group who received family-centered affective stimulation showed significant improvements in cognitive function compared to those in the placebo and control groups. Another study involving both nurses and family members found that patients who received sensory stimulation from either option showed significant improvements in consciousness and cognitive function compared to the control group [84].

**Table 7:** Sensory stimulation therapy in TBI patients.

Study, Year	Sample Size Control/Experimental Group	Method	Cognitive Outcomes	Duration	Motor Outcomes	Other
Moattari et al., 2016 [84]	20/20/20(control/experimental/placebo groups)	Sensory stimulation (auditory, visual, tactile, olfactory)	Gradual increase in cognitive function and basic cognitive sensory recovery especially in family-conducted sensory stimulation group (RLA scale, WNSSP)	7 days, 2 times per day (30 min)	N/A	Improved level of consciousness (GCS)
Salmai et al., 2017 [83]	30/30/30 (family centered/nurse/control groups)	Sensory stimulation (auditory, sensory, kinetic, affective-only in the family centered stimulation)	Enhanced patients’ responsiveness and cognitive functions (CRS-R scores), statistically significant after 4 days	7 days, 2 times per day (30–45 min)	N/A	Statistically significant GCS improvement after 4 days

Legend: CRS-R—Coma Recovery Scale-Revised; GCS—Glasgow Coma Scale; RLA—Rancho Los Amigos; WNSSP—Western Neuro-Sensory Stimulation Profile.

### 3.9. Combined Approaches

Mixed approaches in neurorehabilitation integrate many modalities to meet the varied issues of recovery from TBI. By combining these modalities, patients benefit from increased neuroplasticity due to the synergy of motor and cognitive therapies.

A study conducted by De Luca et al. investigated the effects of combining robotic verticalization training (RVT) with personalized music stimulation in patients diagnosed with chronic minimally conscious state (MCS) due to TBI. The experimental group received music robotic verticalization (MRV) using the Erigo device along with a personalized music playlist, while the control group received only RVT without music stimuli. According to the study, the experimental group significantly improved on a number of measures, such as the Trunk Control Test, Functional Independence Measure, Coma Recovery Scale-Revised, Level of Cognitive Functioning, and Functional Communication Scale [86].

## 4. Discussion

The reviewed studies explored a variety of neurorehabilitation interventions for TBI patients, highlighting cognitive, motor, and psychological outcomes. These interventions included non-invasive brain stimulation techniques (tDCS, rTMS), VR-based therapies, telerehabilitation, RAT, computer-assisted rehabilitation, and behavioral interventions.

Each TBI patient presents with a unique set of challenges, and treatment decisions must be personalized, taking into account the stage of recovery, type, and severity of cognitive or motor deficits, and the patient’s support system. Clinicians should carefully assess these factors to determine the most suitable combination of neurorehabilitation interventions for optimal outcomes. VR-based interventions for TBI patients have demonstrated significant improvements in cognitive functions, particularly in attention, executive function, and visuospatial abilities. VR driving simulations improved working memory and selective attention. VR rehabilitation also led to improvements in balance and mobility. Importantly, VR interventions were well-tolerated, with no reported adverse effects, and many participants maintained cognitive improvements for at least one month post-treatment. Computer-assisted cognitive rehabilitation has significantly improved cognitive function, self-care, and social cognition in moderate to severe TBI patients. Robot-assisted motor therapy programs have shown significant potential in improving motor outcomes, functional mobility, and, in some cases, cognitive outcomes for patients recovering from TBI. Lokomat, one of the most studied robotic devices, demonstrated improvements in walking velocity and gait training across different studies. Non-invasive brain stimulation techniques, such as rTMS and tDCs, have shown varying outcomes in cognitive and motor recovery for patients with TBI. These approaches have targeted executive function, working memory, response inhibition, and motor planning. rTMS studies demonstrated mild improvements in executive function and cognitive processing speed, particularly in tasks such as the Trail Making Test (TMT-B). However, long-term improvements were not consistently observed across evaluations. Short-term cognitive gains were evident in attention and memory, but between-group differences in executive function were not statistically significant. tDCS studies revealed promising results in memory enhancement, working memory and verbal recall. Cognitive improvements from tDCS and rTMS were generally maintained over short follow-up periods, but evidence for sustained benefits beyond a few months is limited. Some studies indicated that behavioral improvements in attention and reaction time remained stable at one-month follow-up, whereas motor gains were less consistent. Telemonitoring and telephone-based studies reported valuable improvements; however, they were not sustained after several weeks Lastly, PBM is an emerging therapy for TBI that uses red and near-infrared light. It shows potential for long-term benefits in improving cognitive function, mood, and overall well-being, with sustained effects observed in memory, attention, and emotional regulation beyond the treatment period. Studies on sensory stimulation have shown that family-centered interventions, in particular, led to significant improvements in TBI patients. However, these studies focused especially on the Glasgow Coma Scale (GCS) scores of comatose TBI patients.

The duration and frequency of interventions varied significantly, ranging from single-session pilot studies to extensive multi-week or even multi-year programs. Despite significant methodological limitations, such as small sample sizes and variation in study designs, these results show the possibility for multimodal neurorehabilitation techniques to optimize recovery in TBI patients.

There are several systematic reviews available on rehabilitation for TBI; however, most of these reviews focus exclusively on a single tool or intervention [26,87,88]. This restricted emphasis limits knowledge of the relative efficacy of various strategies and the possibility of combining several methods to improve recovery results. Previous work by Bonanno et al. highlighted the growing role of innovative technologies in the rehabilitation of patients with TBI, emphasizing their positive impact on cognitive and motor outcomes. Their review underlined the potential clinical benefits of integrating advanced tools into neurorehabilitation practice but called for further studies to establish clearer evidence, particularly in severe TBI, to determine the extent to which these patients may benefit from such interventions [40] This aligns with our aim to provide an updated synthesis of neurorehabilitation strategies, focusing on innovative approaches and their potential for cognitive and motor recovery in TBI populations. Aulisio et al. have discussed VR games as an important tool in neurorehabilitation, focusing especially on movement and motor skills. All investigations on gait and balance found moderate improvement for persons with TBI of various severity. Additionally, studies on upper limb function neurorehabilitation provided mixed results for TBI patients. Significant cognitive rehabilitation has also not been demonstrated in this review [89]. The systematic review by Alashram et al. also investigated VR technology, with a focus on cognitive enhancement. The primary results demonstrated that VR interventions with different treatment protocols are beneficial in enhancing many elements of cognitive function in TBI patients, including memory, executive function, and attention [87]. Another systematic review investigated telerehabilitation as a tool for adult TBI patients. In four out of five randomized controlled trials investigated, scheduled telephone support improved outcomes for persons with mild and moderate-to-severe TBI compared to conventional treatment. However, these studies did not demonstrate or analyze the long-term impacts. There were also no important differences between the control and experimental treatments in the studies that examined two distinct Internet-based therapies that addressed memory and fatigue [90].

There are still a number of gaps in the literature, despite the increased interest in neurorehabilitation for TBI. First, the reliability of the results is constrained by the diversity of intervention methods, small sample sizes, and research design heterogeneity. Second, while many studies demonstrate short-term benefits, there is a lack of evidence on the long-term efficacy and sustainability of these interventions. Furthermore, few studies examine multimodal therapy or directly compare interventions, making it difficult to determine the relative efficacy of various neurorehabilitation techniques. Finally, the lack of consistent outcome measures across research makes it difficult to fully comprehend their effects, especially in the cognitive and psychological domains, which are crucial for TBI recovery.

## 5. Conclusions

This review describes the potential applications of diverse neurorehabilitation strategies in supporting cognitive, motor, and psychological rehabilitation for TBI patients. Various technological innovations and non-invasive approaches have been explored, with reported cognitive benefits related to attention, executive function, and memory. In contrast, motor rehabilitation outcomes demonstrated variability, with some studies reporting limited or inconsistent improvements. While these approaches hold promise, important gaps remain to be discussed, including the lack of long-term data, small sample sizes, and inconsistent outcome measures, reinforcing the importance of conducting additional studies to develop and improve these interventions for the best possible patient recovery.

## Data Availability

No new data created.

## Supplementary Materials

The following supporting information can be downloaded at: https://www.mdpi.com/article/10.3390/life15030503/s1, Table S1: Overview of the included studies.
