# Supplementary material for: Efficacy of Neurorehabilitation Approaches in Traumatic Brain Injury Patients: A Comprehensive Review
Source: Life (Basel). 2025 Mar 20;15(3):503. doi: 10.3390/life15030503 (PMC11944267; doi:10.3390/life15030503)
Supplement: Supplementary file 1 [file life-15-00503-s001.zip › life-3478026-supplementary.pdf]

Study, year	Country	Sample size control/experimental group	Study type	Method	Cognitive Outcomes	Duration	Motor Outcomes	Other
Bell et al, 2017	USA	178/178	RCT	telephone-delivered problem-solving treatment	Experimental group had short-term benefits for psychological distress, sleep quality, depression, PTSD symptoms, and physical health compared to control group ($p<0.05$) However, these improvements were not sustained at 12 months.	Baseline, 6 months, 12 months	N/A	Participants in the experimental group reported higher satisfaction with the intervention and perceived it as more helpful in addressing their challenges
Carneiro et al, 2019	Brasil	10	Prospective observational clinical study	Transcranial PBM	Sustained gains in visuospatial ability and planning, improved processin	18 sessions, delivered three times per week for six weeks.	N/A	improved cerebral bloodflow

					g speed and divided attention, modest improvement in inhibition and selective attention			
De Luca et al, 2023	Italy	10/10	Pilot study	standard cognitive rehabilitation/ virtual reality rehabilitation system	The VR group demonstrated significant improvements in cognitive functions, emotional well-being, and coping strategies compared to the standard rehabilitation group, particularly in attention, executive functioning, and problem-solving	3 months standard neurorehabilitation (6 weekly sessions of 60 min) 3 months advanced rehabilitative (3 weekly sessions of 60 min)	N/A	N/A
De Luca	Italy	8/8	Pilot study	Erigo device for verticalizati	N/A	Three times a week, for about 8 consecutive weeks,	In the experimental	N/A

et al, 2022				on plus music stimulation		each session lasting about 45 min	group, significant changes were found in patients' awareness, global functional outcome, and non-verbal skills, while the control group, showed improvements in the individual scores without reaching a statistical significance.	
De Luca	Italy	15/15	Pilot study	standard cognitive rehabilitati	The experimental	First phase: 3 times a week for 8 weeks	N/A	N/A

et al, 2022				on/ Virtual Reality Based-Attention Processes Training (VB_APT)	group showed significantly greater improvements in global cognition, attention, and depression compared to conventional rehabilitation; significant improvements in executive, visuo-spatial, and attention subtests	Second phase: 24 sessions of 60 min each, 3 times a week for 8 weeks		
Esquenazi et al, 2017	USA	7/8/7 (all interventional)	Randomized, prospective	G-EO; Lokomat; manual assisted BWSTT	N/A	18 sessions of gait training for 6 to 8 weeks, generally 3 times per week. Each session lasted up to 75 min	Functional mobility improved significantly in the G-EO and PBWSTT groups	All three interventions (G-EO, Lokomat, and PBWSTT) significantly improved self-

								selected velocity, but only Lokomat and PBWSTT improved maximal velocity; Improvements in the stroke impact scale mobility domain were seen only in the Lokomat and PBWSTT groups
Ettenhofer et al, 2019	USA	6/11	Pilot study	VR driving simulator NEUROdrive	Intervention group had greater improvement in in working	Six 90-minutes session, 4 weeks	No significant change between group	Greater improvement in intervention group

					memory (p = 0.004); also in visual search/selective attention (p = 0.01) No significant differences between groups in other cognitive measures		s (p > 0.05) in VR tactical and operational scores Scroes remained 'average' at both time points in the intervention group	(p < 0.05) for physical functioning no significant difference (p > 0.05) for mental functioning
Flint et al, 2023	USA	3/3	Experimental study	high-γ/μ–β signals	Motor planning, learning, and adaptability improved in the experimental group	N/A	Motor output (force production), thumb compliance, and physical execution of tasks improved	Differences in success rates between participants (T1: 28% vs. T2: 99%)
Henderson et al, 2017	USA	39	Retrospective	Multi-Watt Near-Infrared Phototherapy	N/A	Each session lasted 30 minutes, with 9–12 minutes of application per target area (8 to 34 sessions/participant)	N/A	Significant improvement in mood and reduced depres

								sion symptoms, reduction in fatigue and enhanced overall well-being
Hipskind et al, 2018	USA	12	Bbservational cohort study	Pulsed Transcranial Red/Near-Infrared Light Therapy Using LED	Significant improvement in symbol search, coding, processing speed; Six of the 15 neuropsychological scales showed significant improvement ($p < 0.05$), particularly in verbal memory and processing speed	20 minutes per session, 3 times per week for 6 weeks (total of 18 sessions)	N/A	N/A
Lee et al, 2018	South Korea	6/7	RCT	neurodevelopmental therapy and rTMS intervention	The experimental group demonstrated significan	30-minute session 5 days per week for 2 weeks	N/A	N/A

					t improvements in post-intervention scores for the Montgomery-Asberg Depression Rating Scale (MADRS), Trail Making Test (TMT), and Stroop Color Word Test; the control group showed no significant changes in these measures.			
Li et al, 2019	UK	24/31	Randomized double blind study	tDCS sessions	Anodal tDCS did not improve response inhibition in TBI patients;control showed significant improvement in	Single visit, three stimulation conditions	N/A	N/A

					SSRT under anodal tDCS. TBI participants showed no significant improvement in stop signal delay under any stimulation condition			
Lin et al, 2023	Taiwan	15/15		Intravenous PBM	Improved short-term memory and attention, reduction in confusion and disorganized behavior	60 minutes session performed on weekdays over two consecutive weeks for each of the three courses	no significant change in motor outcomes	N/A
Liu et al, 2023	USA	13/7	Pilot study do	Participants used instrumented glove on their dominant had and performed a grasp-and-place maneuver; they also	N/A	One single session, three block of trials	Multimodal feedback was effective in enhancing neural activity and impro	N/A

				experienced VR environment			ving motor performance in TBI participants, as demonstrated by increased EEG power, improved motion pathlength, and greater EMG-EEG coherence Neurotypical participants responded better to unimodal feedback, with faster task completion and	

							reduced EMG coherence	
Liu et al, 2021	China	30/30	Clinical trial	computer-assisted cognitive rehabilitation (CACR system)	significantly better cognitive scores in social cognitive ability, self-care ability, sphincter control, and comprehensive ability compared to the control group ($p<0.05$)	N/A	N/A	N/A
Maggio et al, 2019	Italy	28/28	Retrospective CCS	Lokomat Pro, equipped with a VR screen/Lokomat Nanos, without VR	Significant improvement in global cognitive function, cognitive flexibility and shifting skills, selective attention and visual search for experimental group group.	40 one-hour sessions (8 weeks, 5 times/week)	Experimental group showed significant improvement in executive functions Both groups showed significant	Experimental group showed significant improvement in overall quality of life

					Both groups showed significant improvement in mood well-being		improvement in physical well-being	
Moattari et al, 2016	Iran	20/20/20(con-trol/experimen-tal/placebo groups)	RCT	Sensory stim-ulation (audi-to-ry,visual,tactile,olfactory)	Gradual increase in cogni-tive function and basic cognitive sensory recovery especially in fami-ly-conducted sensory stimulation group (RLA scale, WNSSP)	7 days, 2 times per day (30 minutes)	N/A	Improved level of consciousness (GCS)
Motes et al, 2019	USA	6/8	Prospective	HD-tDCS sessions	significant differences between the active and sham groups in the changes in the total score of the Rey Auditory Verbal Learning	10 sessions of 20 minutes of 1 mA anodal HD-tDCS	N/A	N/A

					Test (RAVLT), the time taken for the Delis-Kaplan Executive Function System (DKEFS) Inhibition test, and the time taken for the DKEFS Inhibition/Switching test			
Neville et al, 2019	Brazil	13/17	Randomized, double blind	rTMS was applied using a magnetic stimulator (MagPro X100)	N/A	5 seconds of rhythmic high-frequency, dayly for 10 sessions	No consistent improvement in executive function of controls versus patients	N/A
O'Neil-Piroz et al, 2017	USA	4/4	Pilot study	tDCS sessions	For the TBI group, word recall increased in the anodal condition for all participa	three 90-minute sessions, a minimum of 48 hours apart	N/A	P300 latency increased across all conditions for the

					nts (+3 to +6 words) and in the sham condition for two participants			TBI group
Quinn et al, 2020	USA	14/10	Pilot study	computerized executive function training combined with TDCS	Participants demonstrated significant improvements in depression, anxiety, post-concussive symptoms, complex attention, and executive functions from baseline to post-treatment visits ($p < 0.01$)	30 minutes sessions, 10 weekdays	N/A	Global cerebral blood flow decreased significantly from baseline to post-treatment visits ($p = 0.02$), with no significant differences between mild and moderate TBI participants.
Raso et al, 2021	Italy	11/11	Pilot study	Scheduled videoconferences patient-clinical	Higher mortality in the LSH program	N/A	4 years of intervention period	Lower daily health care costs

				unit; wearable monitoring devices	(36%) compared to the telemonitoring group (18%). Bedsores (18% vs. 0%) and infections (36% vs. 18%) were more common in the LSH group, but differences were not significant. No significant differences between groups in neuropsychological scores		, with monthly consultations for the telemonitoring group	
Rodriguez-Rajo et al, 2024	Spain	26/28	Quasi-RCT	computerized tasks module designed for the rehabilitation of social cognition	Experimental group showed better results for almost all measures Improved ability	Weekly sessions	N/A	N/A

					to recognize facial emotions in the control group Experimental group demonstrated better ability to recognize emotions or mental states from eyes post-treatment compared to the control group.			
Sacco et al, 2016	Italy	16/16	Pilot study	tDCS stimulation (HDCstim device)	within the experimental group, significant improvements were observed between pre-training and post-training , with faster reaction times (p =	Ten sessions, each session included 20 min of tDCS stimulation followed by 30 min of cognitive training	N/A	N/A

					0.004) and fewer omission error; he control group did not exhibit any significant changes; d borderline improvement in attention performance (p = 0.057) within the experimental group, although no significant changes were found in visual-spatial abilities, semantic fluency, working memory, or long-term memory			
Salmani	Iran	30/30/30 (family cen-	RCT	Sensory stim-ulation	Enhanced patients'	7 days, 2 times per day (30-45 minutes)	N/A	Statistically signifi

et al, 2017		tered/nurse/control groups)		(audi-tory, sensory, kinetic, affec-tive-only in the family cen-tered stimula-tion)	responsiveness and cognitive functions (CRS-R scores), statistically significant after 4 days			cant GCS improvement after 4 days
Teter filler et al., 2019	USA	32/31	RCT	Xbox Kinect games	N/A	3–4 times per week for 12 weeks, lasting for 30 min.	no statistically significant difference between the two groups, both treatment groups showed improved balance responses to these therapies	N/A
Vuletic et al, 2016	USA	178/178	RCT	control group: mailing of educational brochures experimental group:	N/A	Biweekly over a 6 months period	N/A	Experimental group showed significant

				telephone calls				improvements in overall sleep quality, sleep duration, latency, and habitual sleep efficiency at 6 months. Improvements in sleep were not sustained at 12 months.

Table S1. Overview of the included studies
